# Reducing soft-tissue shrinkage artefacts caused by staining with Lugol’s solution

**DOI:** 10.1038/s41598-021-99202-2

**Published:** 2021-10-05

**Authors:** Y. Dawood, J. Hagoort, B. A. Siadari, J. M. Ruijter, Q. D. Gunst, N. H. J. Lobe, G. J. Strijkers, B. S. de Bakker, M. J. B. van den Hoff

**Affiliations:** 1 Obstetrics and Gynaecology, Amsterdam Reproduction and Development Research Institute, Amsterdam UMC, University of Amsterdam, Meibergdreef 9, Amsterdam, The Netherlands; 2 Medical Biology, Amsterdam UMC, University of Amsterdam, Meibergdreef 15, Amsterdam, The Netherlands; 3 Biomedical Engineering and Physics, Amsterdam UMC, University of Amsterdam, Meibergdreef 9, Amsterdam, The Netherlands; 4 Radiology, Amsterdam UMC, University of Amsterdam, Meibergdreef 9, Amsterdam, The Netherlands

**Keywords:** Developmental biology, Anatomy, Medical research

## Abstract

Diffusible iodine-based contrast-enhanced computed tomography (diceCT) is progressively used in clinical and morphological research to study developmental anatomy. Lugol’s solution (Lugol) has gained interest as an effective contrast agent; however, usage is limited due to extensive soft-tissue shrinkage. The mechanism of Lugol-induced shrinkage and how to prevent it is largely unknown, hampering applications of Lugol in clinical or forensic cases where tissue shrinkage can lead to erroneous diagnostic conclusions. Shrinkage was suggested to be due to an osmotic imbalance between tissue and solution. Pilot experiments pointed to acidification of Lugol, but the relation of acidification and tissue shrinkage was not evaluated. In this study, we analyzed the relation between tissue shrinkage, osmolarity and acidification of the solution during staining. Changes in tissue volume were measured on 2D-segmented magnetic resonance and diceCT images using AMIRA software. Partial correlation and stepwise regression analysis showed that acidification of Lugol is the main cause of tissue shrinkage. To prevent acidification, we developed a buffered Lugol’s solution (B-Lugol) and showed that stabilizing its pH almost completely prevented shrinkage without affecting staining. Changing from Lugol to B-Lugol is a major improvement for clinical and morphological research and only requires a minor adaptation of the staining protocol.

## Introduction

Over the last decades, three-dimensional (3D) imaging techniques greatly improved morphological research by enabling non-destructive visualization of anatomical and morphological structures at a histological level^1^. Diffusible iodine-based contrast-enhanced computed tomography (diceCT) is a major scientific breakthrough and is progressively used in morphological research^2^. Currently, diceCT with the use of microfocus machinery (i.e. micro-CT) is also being evaluated in a clinical setting for post-mortem fetal imaging as adjunct, or even replacement, of autopsy^2–4^. DiceCT, alongside Ultra-High Field Magnetic Resonance Imaging (UHF-MRI), enables the study of development of internal structures and their anatomy, including soft tissues^5–7^. Besides its non-destructive character and the acquisition of high-resolution images, specimens once stained using iodine may be used subsequently in histological studies to verify and validate findings^2^.

**Figure 1:**
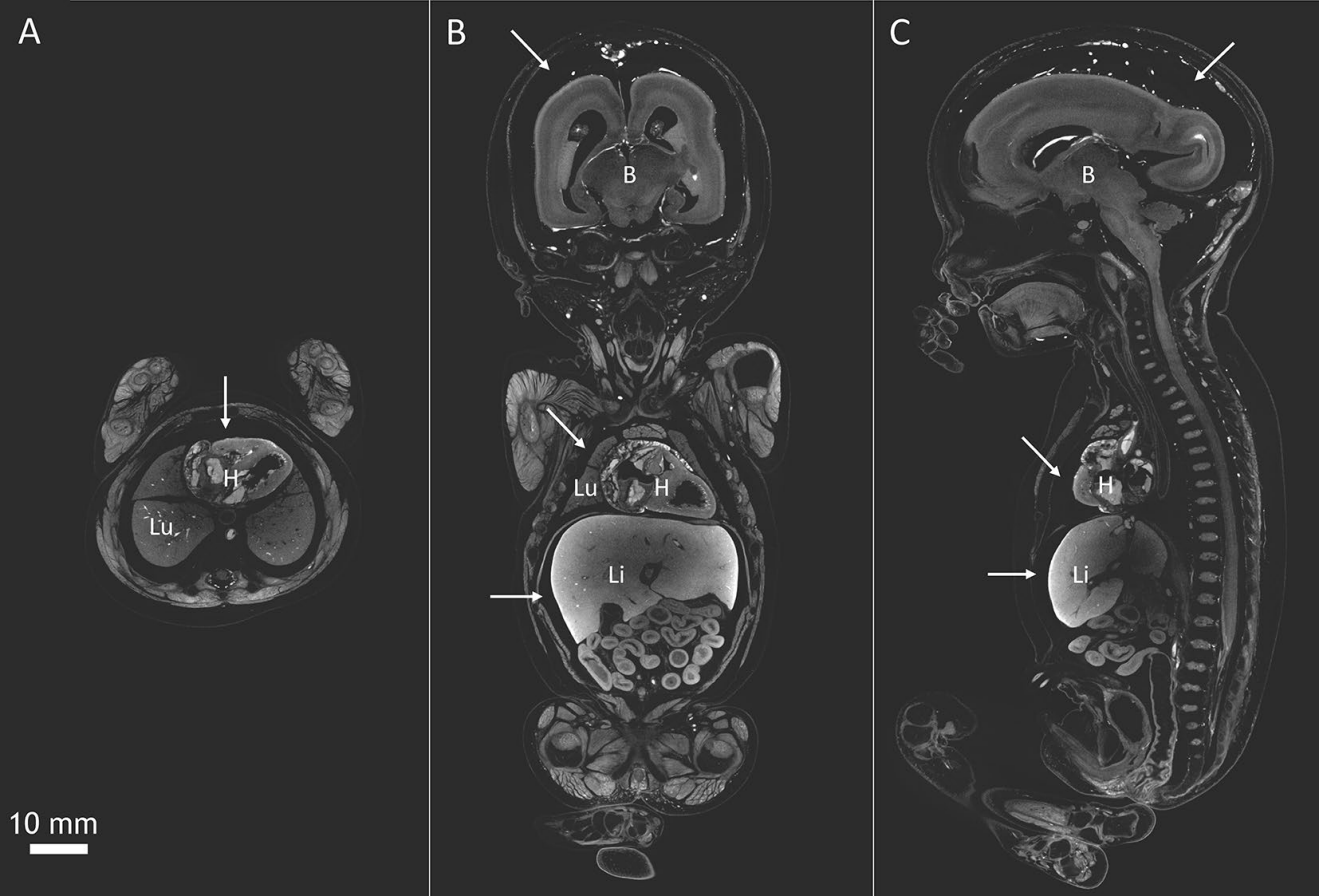
Micro-CT images of a human fetus at 19 weeks of gestation (total length = 24 cm). The fetus was stained with 3.75% Lugol’s solution for 26 days in total. The fetus was scanned on a GE Phoenix v|tome|x scanner (General Electric, Wunstorf, Germany) with an isotropic resolution of 50 µm. (**A**) Transversal section at the level of the heart, (**B**) mid coronal section and (**C**) mid sagittal section. Volume analyses showed that the staining caused extensive shrinkage: 9% in total fetal volume, 24% in lung and liver volume, 31% in kidney volume and 33% in brain volume Images adapted from *Organ specific shrinkage in iodine stained human fetuses*^13^. White arrows point to the empty space between skull and brain and liver and body cavity, which is reminiscent of soft-tissue shrinkage. B = Brain, H = Heart, Li = Liver, Lu = Lung. Scale bar represents 10 mm.

Potassium triiodide (I_2_KI) solution, also known as Lugol’s solution (Lugol), has gained interest as an effective contrast agent because of its relative ease of handling, cost-effectiveness and differential affinity for different types of soft tissue^5,8,9^. An important limitation of using Lugol is that the staining process causes extensive soft-tissue shrinkage^7,10.11^. This shrinkage was shown to be I_2_KI concentration dependent^10,12^ and to vary across tissue types ^10,13^. We have seen extensive shrinkage in lung, brain, liver and total body volume after staining of human fetal specimens using 3.75% Lugol, which varied between 15 to 35% of the initial volume using MR images recorded prior to staining as a reference (Fig. 1)^13^. Such differential shrinkage of fetal tissues might obscure malformations or induce artefacts which erroneously could be interpreted as malformations. The lack of knowledge about how these shrinkage artefacts can be reduced, or prevented, hampers future application of Lugol in morphological research, especially in clinical or forensic cases where tissue shrinkage can lead to erroneous diagnostic conclusions.

The mechanism of the Lugol-induced differential tissue shrinkage is largely unknown but was suggested to be due to an osmotic imbalance between tissue and solution^11^. However, the use of isotonic^11^ or even hypotonic Lugol^11,13^ was not found to prevent tissue shrinkage, thus demonstrating that osmotic imbalance cannot be the sole mechanism. Pilot experiments to evaluate different Lugol’s solutions used in the staining procedure point to acidification of the solution, rather than changes in osmolarity as cause of tissue shrinkage. Although acidification has been suggested to cause demineralization of bone tissue, its relation with tissue shrinkage has never been evaluated^14^.

In this study, we used mouse livers to assess the relation between osmotic imbalance, acidification of Lugol and tissue shrinkage over time. The results showed that acidification of Lugol is the main factor affecting tissue shrinkage. Hence, we hypothesized that stabilizing the pH of the staining solution, thus preventing its acidification, would reduce or even prevent tissue shrinkage. We, therefore, designed a modified Lugol’s solution, referred to as buffered Lugol’s solution (B-Lugol), and measured pH and tissue shrinkage by staining an additional series of mouse livers and compared this to Lugol. Following the successful application of B-Lugol on mouse livers, we stained and imaged three human fetal samples. These specimens provided evidence that this new formula is also suitable to stain complex organisms with minimal tissue shrinkage.

## Results

### Mouse liver experiments

To study the amount of tissue shrinkage due to staining with Lugol’s solution (Lugol) and to determine the contribution of osmolarity and pH to this shrinkage, a total of 15 mouse livers (N = 3 per condition) were stained in five different solutions of Lugol with different concentration and/or tonicities (i.e. five conditions); while the liver volume, as well as the osmolarity and the pH of the staining solution were monitored over time. As controls, three mouse livers were not stained and remained in the storage solution (0.2% PFA in PBS). Because initial volumes of the livers differed, systematic differences in observed liver volumes were corrected by determining and applying a correction factor per liver^15^ and normalized by setting the mean volume at time 0 to 100% (Table 1).

**Table 1:** Between-liver correction and normalization.

Experiment 1				Experiment 2			
Condition	Livers	Between-liver correction	Normalization (time 0 = 100)	Condition	Livers	Between-liver correction	Normalization (time 0 = 100)
0.2% PFA in PBS	1	1.114	421.78	3.75% Lugol	19	1.072	518.51
	2	0.955			20	1.153	
	3	1.031			21	0.955	
Hypotonic 1.25%	4	1.188	453.50		22	0.775	512.41
	5	0.733			23	0.844	
	6	1.031			24	1.032	
Hypotonic 2.5%	7	1.128	571.64		25	1.283	489.17
	8	0.994			26	0.775	
	9	1.458			27	0.862	
Isotonic 1.25%	10	0.786	426.48	3.75% B-Lugol	28	1.047	443.82
	11	1.133			29	1.141	
	12	0.932			30	0.828	
Isotonic 2.5%	13	0.868	533.47		31	1.319	435.30
	14	0.818			32	0.990	
	15	1.691			33	0.794	
Isotonic 3.75%	16	1.025	401.51		34	1.052	449.56
	17	0.760			35	1.265	
	18	0.800			36	1.033	
					37	1.077	448.89
					38	0.981	
					39	0.983	

To remove random differences between livers, without losing the effects of Condition and Time, a correction factor (column Between-liver correction) was determined^15^ and applied to the data measured for each liver. Thereafter, the volume data were normalized by dividing each value by the average volume per condition at time 0 (column normalization factor) and multiplied by 100; effectively scaling the average volume at time 0 to 100%.

**Figure 2:**
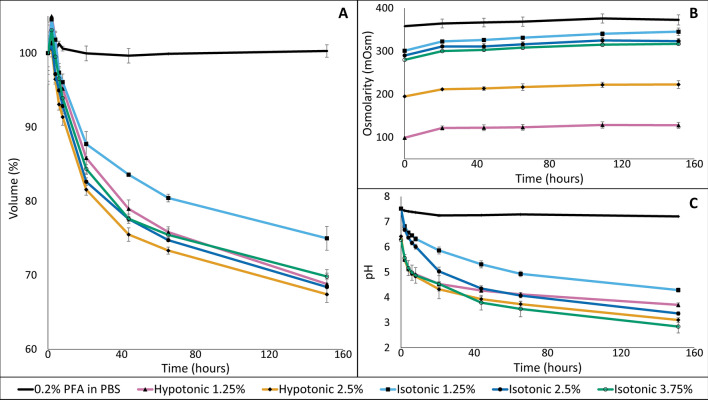
Mouse livers stained in different Lugol’s solutions. Mouse livers were stained in Lugol’s solutions that differed with respect to Lugol concentrations and tonicities. Observed liver volumes were normalized by setting the mean volume at time 0 to 100%. All presented values are the average and standard deviation of three different livers. (**A**) *Liver volume.* Liver volume remained stable in the control samples, with an average increase of 0.3% ± 0.8% relative to its volume at the start of the staining. In Lugo’s solution, irrespective of the Lugol concentration or tonicity, liver volumes decreased at least 25%. (**B**) *Osmolarity*. Independent of the Lugol concentration or original tonicity, the osmolarity of each staining solution was observed to increase over time. The largest increase was observed in the 1.25% isotonic Lugol (44 ± 7 mOsm) and the smallest increase in storage solution (15 ± 10 mOsm). (**C**)* pH.* The pH of the staining solutions decreased significantly over the staining period for all different Lugol solutions, except for the storage solution. The higher the concentration of Lugol, the lower the pH became, with the lowest pH measured in 3.75% isotonic Lugol (pH 2.8 ± 0.2).

The liver volume remained stable for the three control samples, with an average increase of 0.3 ± 0.8% (mean ± SD). For all livers stained in the different concentrations and/or tonicities of Lugol, a significant decrease in volume was observed of at least 25%. The highest amount of shrinkage was observed in 2.5% hypotonic Lugol (32.6 ± 1.1%) and the lowest in 1.25% isotonic Lugol (25.0 ± 1.6%) (Fig. 2A).

The osmolarity of 3.75% Lugol was reported to be isotonic with respect to human and mouse tissue^11^ and after preparation of this solution its osmolarity was confirmed to be approximately 300 milliosmole (mOsm). After preparing the other solutions their osmolarity was measured and found to be at the expected level of 195 mOsm for 2.5% hypotonic Lugol, 99 mOsm for 1.25% hypotonic Lugol, 290 mOsm for 2.5% isotonic Lugol and 301 mOsm for 1.25% isotonic Lugol. Independent of the Lugol concentration, these osmolarities were observed to increase with increasing staining time (Fig. 2B). The largest increase was observed in the 1.25% isotonic Lugol (44 ± 7 mOsm) and the smallest increase in the 0.2% PFA in PBS (15 ± 10 mOsm) after 152 h of incubation (Fig. 2B).

All freshly prepared Lugol’s solutions showed a pH between 6.0 and 7.5. The pH decreased substantially during the staining period for all conditions, except for the control samples (Fig. 2C). In all Lugol’s solutions used, an exponential decrease in pH was observed, steep in the first two hours of staining and more gradual up to the last time point included (152 h). Since pH is a log-based measurement, this exponential decrease of pH over time means that the H^+^-ion concentration increases linearly with the logarithm of staining time. Moreover, the higher the starting concentration of Lugol was, the lower the pH value was, with the lowest pH value measured in 3.75% isotonic Lugol after 152 h (pH 2.8 ± 0.2). Furthermore, the isotonic 2.5% and 1.25% Lugol solutions became less acidic (pH 3.3 ± 0.1 and 4.3 ± 0.1, respectively) compared to the hypotonic 2.5% and 1.25% Lugol solutions (pH 3.1 ± 0.1 and 3.4 ± 0.1, respectively).

**Figure 3:**
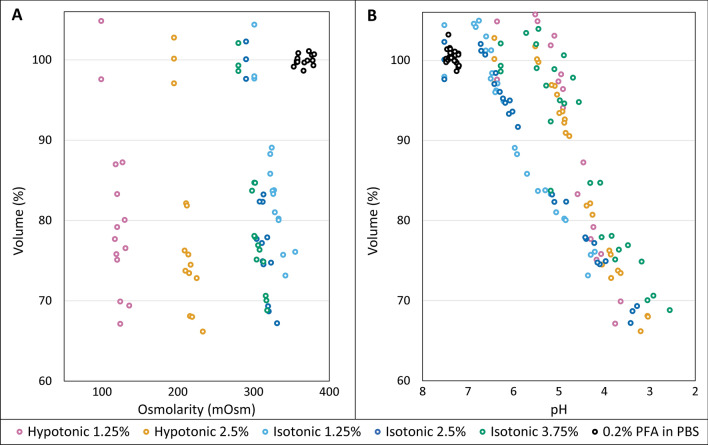
Scatterplots showing the relation between liver volume and osmolarity or pH of the staining solution. Observed liver volumes were normalized by setting the mean volume at time 0 to 100%. Each dot represents a mouse liver at a different time point. (**A**)* Volume versus Osmolarity*. The decrease in tissue volume in isotonic solutions (approximately 300 mOsm) was in the same range as in both hypotonic solutions (approx. 100 and 200 mOsm), showing no correlation between tissue shrinkage and osmolarity. (**B**)* Volume versus pH.* The decrease in tissue volume, tissue shrinkage, shows a similar trend with decreasing pH in each of the tested Lugol’s solutions.

Evaluation of the relation between the tonicity of the different solutions and tissue shrinkage during the staining procedure revealed that the decrease in tissue volume occurred in a similar range in isotonic as well as hypotonic solutions (Fig. 3A). Partial correlation analysis, controlling for the correlation of each variable with time (Fig. 2), showed no significant correlation between osmolarity of the solution and neither tissue volume (r = 0.05, *p* = 0.674) nor pH (r = 1.66, *p* = 0.177). On the other hand, pH and tissue volume were significantly correlated (r = 0.627, *p* < 0.001); in all Lugol solutions, tissue volume showed similar downward trends with decreasing pH (Fig. 3B). Linear regression analysis of volume on pH resulted in R^2^ values between 0.63 and 0.99, indicating that between 63 and 99% of the variation in tissue volume could be explained by the decreasing pH (Table 2A, R^2^ values; Supplemental PDF: SPSS output). Further regression analysis of volume on pH interestingly showed that overlapping confidence intervals for the slope coefficients in each of the solutions, pointing to a similar role of acidification in Lugol-induced tissue shrinkage across conditions (Table 2A; B-coefficients). As expected from the correlation analysis, when the residual volumes were fitted to osmolarity, significant regression coefficients were found in 1.25% and 3.75% Lugol (Table 2A). However, calculation of the contribution of osmolarity to the predicted liver volume showed that on average only 1% to 5% of the residual volume variation could be explained by osmolarity change (Supplementary PDF: SPSS output). Taken together, this stepwise multiple regression analysis showed that the decrease in tissue volume over staining time can primarily be attributed to the decreasing pH, and that the osmolarity of the staining solution only plays a minor, if any, role in causing tissue shrinkage.

**Table 2A:** Stepwise regression analysis in Experiment 1.

Experiment 1						
Step 1: regression of Volume on pH (saving residuals to Res1)						
Model: Volume = A + B * pH						
Condition	R^2^		Coeff. value	Std. Error	95% confidence interval	
					Lower	Upper
Hypotonic 1.25%	0.630	A	34.06	8.75	15.91	52.20
		B	11.02	1.74	7.42	14.62
Hypotonic 2.5%	0.970	A	19.16	2.43	14.13	24.20
		B	14.84	0.54	13.72	15.96
Isotonic 1.25%	0.952	A	22.91	3.21	16.26	29.56
		B	11.67	0.55	10.54	12.80
Isotonic 2.5%	0.990	A	35.21	1.10	32.92	37.49
		B	9.71	0.21	9.28	10.13
Isotonic 3.75%	0.858	A	35.13	4.52	25.75	44.51
		B	11.89	1.00	9.80	13.97

Step 2: regression of Res1 on Osm						
Model: Res1 = A + B * Osm						
Condition	R^2^		Coeff. value	Std. Error	95% confidence interval	
					Lower	Upper
Hypotonic 1.25%	− 0.044	A	12.10	21.98	− 36.88	61.08
		B	− 0.13	0.18	− 0.53	0.27
Hypotonic 2.5%	− 0.109	A	2.85	26.16	− 56.32	62.02
		B	− 0.02	0.12	− 0.29	0.26
Isotonic 1.25%	0.720	A	− 72.69	13.31	− 102.35	− 43.03
		B	0.22	0.04	0.13	0.31
Isotonic 2.5%	− 0.095	A	3.67	18.30	− 37.11	44.45
		B	− 0.01	0.06	− 0.14	0.12
Isotonic 3.75%	0.422	A	− 124.18	45.24	− 224.98	− 23.38
		B	0.40	0.15	0.07	0.73

Multiple regression analysis was performed out in two steps. Firstly, a regression of Volume on pH was carried out, storing the residual in the variable Res1. Secondly, a regression was carried out of Res1 on Osm. For both analyses the R^2^, coefficient value and standard error of the A and B coefficients are given as well as their 95% confidence interval. Note that the intercepts and slope coefficients (A and B) of the volume—pH relation show overlapping confidence intervals among conditions, indicating that the effect of pH on volume is similar in the different conditions.

#### Stabilizing pH reduces shrinkage artifacts

We hypothesized that stabilizing the pH of the staining solution would prevent acidification of the solution and subsequently reduce or even prevent tissue shrinkage. In an effort to stabilize the pH, Lugol was prepared in a strong phosphate buffer (Sorensen’s buffer). In this manuscript, this buffered Lugol’s solution will be referred to as B-Lugol, while unbuffered Lugol’s solution will be referred to as Lugol. To evaluate the effect of this stronger phosphate buffer, mouse livers were stained using 3.75% B-Lugol (N = 12) and compared to mouse livers that were stained using 3.75% Lugol (N = 12).

**Figure 4:**
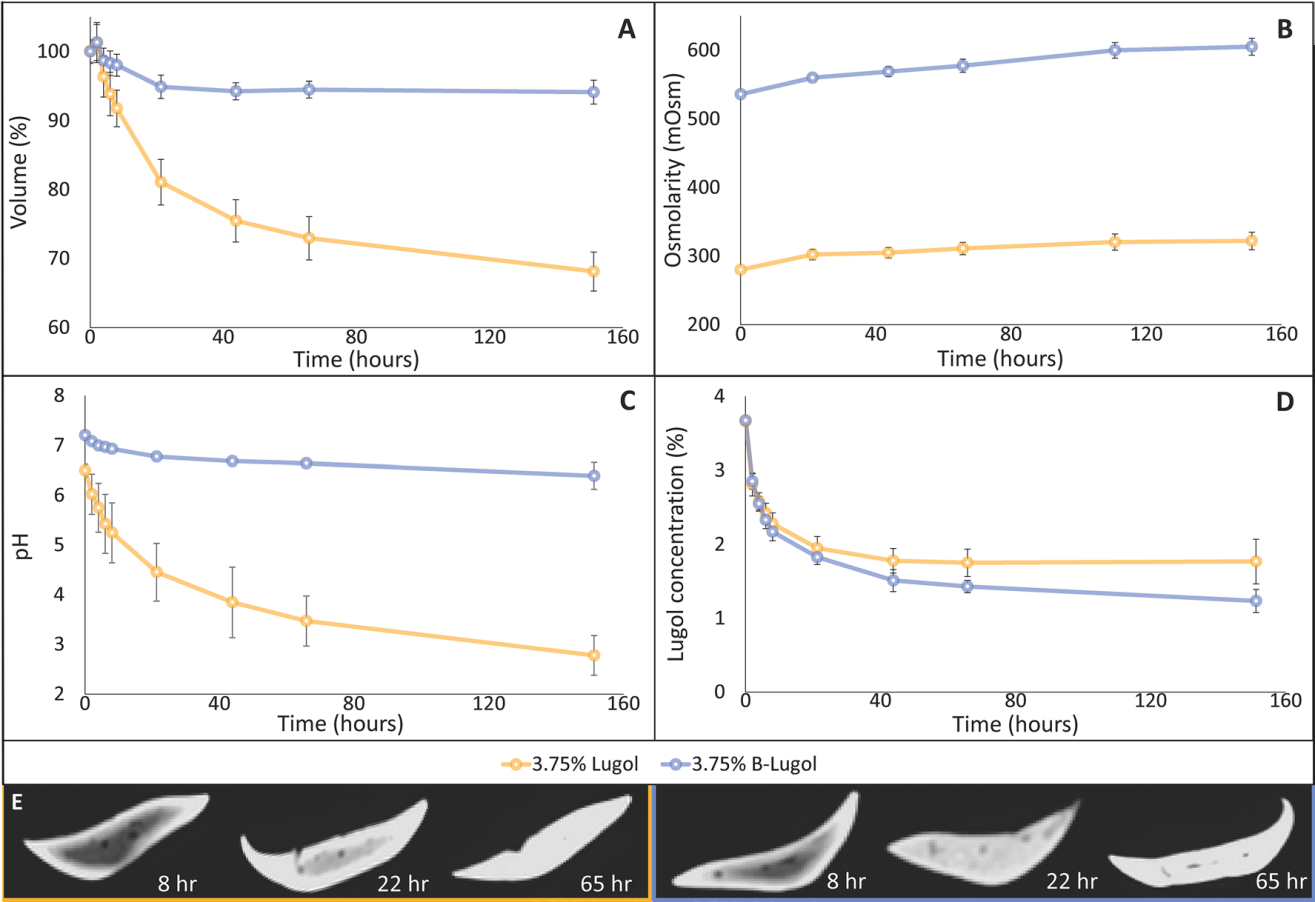
Comparison of staining of livers in 3.75% Lugol and 3.75% B-Lugol. The values are the average and standard deviation of 12 biological replicates. (**A**)* Liver volume.* The volume of the livers in B-Lugol decreased slightly during staining, whereas in Lugol the mouse liver volumes almost decreased to one third of the original volume. (**B**)* Osmolarity.* In both solutions the osmolarity increased over time. (**C**)* pH*. The pH of the Lugol’s solutions decreased significantly in both Lugol and B-Lugol, 6.50 ± 0.1 to 2.8 ± 0.4 and 7.2 ± 0.0 to 6.4 ± 0.3, respectively. However, in B-Lugol this decrease was small because of the buffering. (**D**)* Lugol concentration.* Both solutions showed a similar decrease in the Lugol concentration. (**E**)* Tissue staining.* The binding of triiodide to the tissue is illustrated in 2D CT images of the mouse livers after 8, 22 and 65 h of staining (red outlined = stained in Lugol, green outlined = stained in B-Lugol), showing comparable staining intensity and penetration over time.

During the 152 h staining period the livers incubated in 3.75% Lugol showed a decrease in volume of 31.9 ± 2.8%, which is in line with the previous experiment. The volumes of the livers incubated in 3.75% B-Lugol decreased significantly  (5.9 ± 1.7%; *p* < 0.001) over the complete staining period, but only slightly compared to 3.75% Lugol (Fig. 4A). In the presence of Sorenson’s buffer, the pH showed a decrease from 7.2 ± 0.0 to 6.4 ± 0.3 (Fig. 4C). However, this decrease is small compared to the decrease in pH of 3.75% Lugol, which decreased from 6.5 ± 0.1 to 2.8 ± 0.4 (Fig. 4C). Regression analysis showed that these pH changes were enough to explain 57% and 90% of the variation in tissue volume in 3.75% B-Lugol and 3.75% Lugol, respectively (Table 2B, R^2^ values). As in the first experiment, the confidence intervals of the slope parameters overlapped between 3.75% B-Lugol and 3.75% Lugol (Table 2B), and also with those confidence intervals observed in the five solutions of the first experiment (Table 2A). This shows that the role of pH on tissue shrinkage is similar in both experiments is. Because of the small decrease of the pH in B-Lugol hardly any tissue shrinkage is observed in this solution (Fig. 4A). As expected, the osmolarity of 3.75% B-Lugol was hypertonic (536 mOsm) at the start of staining and increased 69 ± 12 mOsm over time, showing a larger increase compared to the osmolarity of 3.75% Lugol (42 ± 13 mOsm) (Fig. 4B). However, the regression analysis showed no significant contribution of this osmolarity change to the variation in tissue volume (Table 2B).

**Table 2B:** Stepwise regression analysis in Experiment 2.

Experiment 2						
Step 1: regression of Volume on pH (saving residuals to Res1)						
Model: Volume = A + B * pH						
Condition	R^2^		Coeff. value	Std. Error	95% confidence interval	
					Lower	Upper
3.75% Lugol	0.899	A	44.556	1.609	41.35	47.76
		B	8.485	0.317	7.85	9.12
3.75% B-Lugol	0.570	A	24.33	6.12	12.19	36.47
		B	10.610	0.892	8.84	12.38

Step 2: regression of Res1 on Osm						
Model: Res1 = A + B * Osm						
Condition	R^2^		Coeff. value	Std. Error	95% confidence interval	
					Lower	Upper
3.75% Lugol	0.010	A	3.55	7.29	− 11.16	18.26
		B	− 0.02	0.024	− 0.064	0.033
3.75% B-Lugol	0.110	A	− 15.87	5.38	− 26.64	− 5.09
		B	0.03	0.01	0.008	0.046

For description of the procedure: see Table 2A. Note that the intercepts (A) and slope coefficients (B) of the volume—pH relation show overlapping confidence intervals with those in the different conditions in Experiment 1, indicating that, even in B-Lugol a similar effect of pH on volume is present, but hardly any volume change is observed because the pH is almost constant.

#### Triiodide uptake is similar in unbuffered and buffered Lugol’s solution

By changing the buffer system of the Lugol’s solution, the triiodide uptake into the tissue might be altered. The triiodide uptake in the tissue was determined from the decrease of triiodide concentration in the solution, measured spectrophotometrically in the solution at different time points (Fig. 4D). To quantify the concentration of triiodide in the staining solution, a calibration curve of optical density (OD) versus defined Lugol concentrations was prepared by serial dilution of the Lugol’s stock solution (Supplemental Fig. 1). Figure 4D shows that the decrease in Lugol concentration is similar between 3.75% Lugol and 3.75% B-Lugol, suggesting that changing the buffer system does not negatively affect the triiodide uptake in the mouse livers. This analysis also showed that the largest decrease in the concentration of Lugol, and thereby the fastest uptake of triiodide in the tissue, occurs in the first 24 h of staining in both solutions. Between 40 and 152 h the Lugol concentration only decreased slightly, suggesting saturation of the uptake of triiodide in the livers. These observations are in line with the observations in 2D CT images, showing that the staining intensity gradually increases from the periphery to the center of the tissue. The observation that in both Lugol and B-Lugol the center of the liver was similarly stained after 65 h (Fig. 4E) is highly suggestive of a similar uptake velocity of triiodide in the tissue in unbuffered Lugol and buffered B-Lugol, and that triiodide uptake is therefore considered independent of the buffer system.

### Human fetus experiments

Three human fetuses were stained in B-Lugol to provide evidence that this new formulation of Lugol is also suitable to stain complex specimens and to show that B-Lugol prevents differential shrinkage that occurs when Lugol is used for staining. As a starting reference for the volume of the entire fetus and its organs, an MRI scan was made because a CT scan of unstained tissue hardly shows any anatomical detail at this developmental stage. During the B-Lugol staining period CT scans were made at different time points and, in these images, volume of the body, the brain, the lungs, the liver and the kidneys were measured.

**Figure 5:**
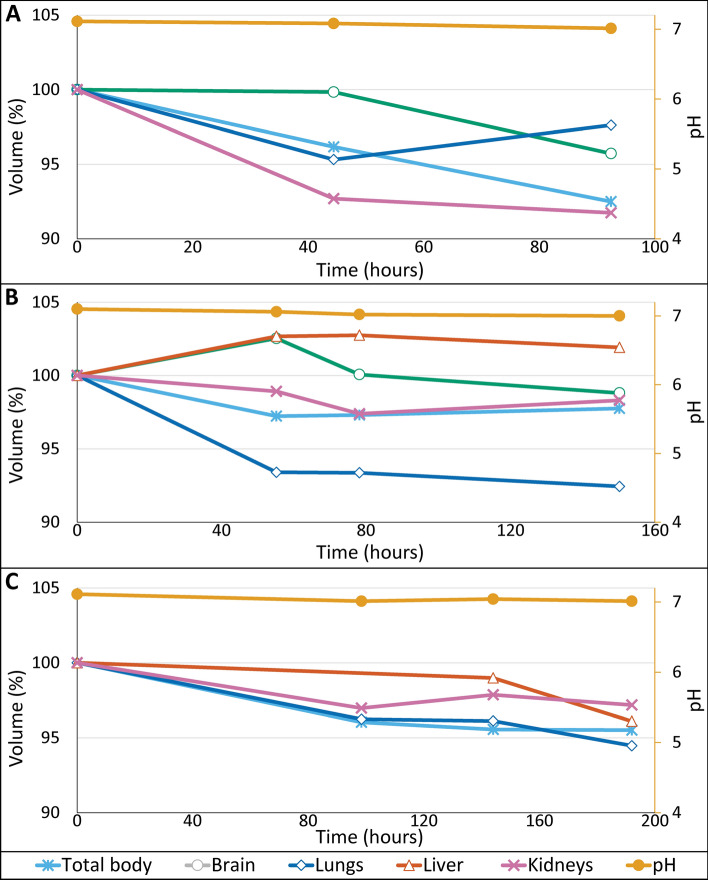
Three different human fetuses stained in 3.75% B-Lugol for a period of 90 to 190 h depending on fetal size. pH remained stable (red line) with a slight decrease from 7.1 to 7.0 (right Y-axis) during the entire staining procedure. (**A**) Fetus #1, 13 + 1 weeks of gestation (weeks + days), total length = 8 cm, weight = 17 g. (**B**) Fetus #2, 15 + 2 weeks of gestation, total length = 15 cm, weight = 58 g). (**C**) Fetus #3, 16 + 2 weeks of gestation, total length = 17 cm, weight = 85. In each of the fetuses, total body volume and almost all organ volumes showed only a limited shrinkage of between 0 and 5%. Only the total body and kidneys of fetus #1 (**A**) and lungs in Fetus #2 (**B**) showed, with 8%, slightly more shrinkage.

During the entire staining procedure, the pH of B-Lugol remained, as expected, stable with a slight decrease from 7.1 to 7.0 for all three fetuses (Fig. 5, right Y-axis). In line with the mouse liver experiments, the osmolarity of the 3.75% B-Lugol gradually increased and the Lugol concentration (OD measurements) decreased over time (data not shown). Analysis of the CT scans showed that all internal organs were completely and uniformly stained after 92, 150 and 192 h for fetus #1, #2 and #3, respectively. The observed difference in staining time is correlated with specimen size; the larger the specimen, the longer the required staining time. Measuring the volume of the fetus and its organs revealed that there was a small degree of shrinkage, which showed some organ-specificity. In general, the measured volumes revealed a decrease of at most 5% compared to the original volume measured in the MRI images (Fig. 5, left Y-axis). However, it is of relevance to note that the volume of the total body and kidneys of fetus #1 (Fig. 5A) and the lungs of fetus #2 (Fig. 5B), were affected most, showing a decrease of 8% after the entire staining procedure.

**Figure 6:**
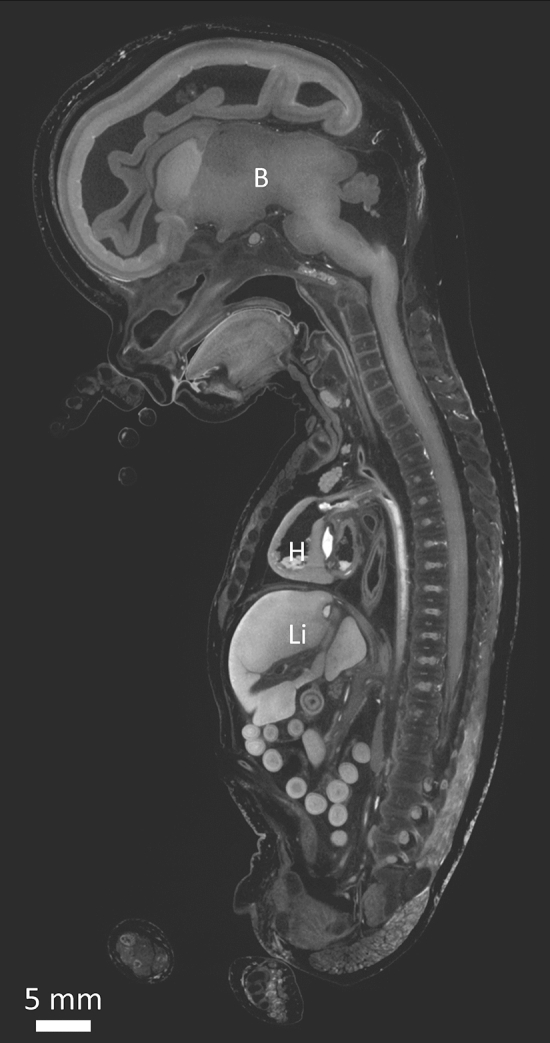
Fetus #2 stained with 3.75% B-Lugol. Mid sagittal section of fetus #2 (15 + 2 weeks of gestation (weeks + days), total length = 15 cm, weight = 58 g). It was stained for 150 h in 3.75% B-Lugol, image analysis shows hardly any shrinkage reflected by the almost no space around the organs, as compared to the fetus shown in Fig. 1 which was stained in Lugol. B = Brain, H = Heart, Li = Liver. Scale bar represents 5 mm.

To illustrate the beneficial effect of B-Lugol compared to Lugol, Fig. 6 shows a micro-CT image of a 15 + 2 weeks old fetus (fetus #2), stained with 3.75% B-Lugol. The 19 weeks old fetus shown in Fig. 1, was fixed identically, stained similarly (in 10 volumes relative to the fetal weight)  3.75% Lugol and scanned at the same time points. Compared to the fetus in Fig. 6, the fetus stained in Lugol, showed extensive shrinkage: 9% in total fetal volume, 24% in lung and liver volume, 31% in kidney volume and 33% in brain volume. This differential shrinkage is reflected in the increase of empty space between skull and brain and liver and body cavity. In contrast, upon staining in B-Lugol hardly any shrinkage was observed in fetus #2, with at most 8% shrinkage of the lungs (Fig. 5B); note that hardly any empty space is observed between the skull and the brain, nor the liver and body cavity (Fig. 6).

## Discussion

In this study we show that acidification, rather than osmolarity, is the key player underlying soft-tissue shrinkage that occurs during Lugol staining. Based on this finding we adapted the staining protocol such that tissue shrinkage was reduced to a level that is within the biological variation among fetuses of the same age (c.f. fetus #2 and #3).

Our experiments showed that, in line with published research, the use of Lugol’s solution causes extensive tissue shrinkage^10,11^. However, contrary to earlier hypotheses^10–12^ we did not observe a significant correlation between osmolarity and tissue shrinkage: the same range of shrinkage occurred in hypotonic as well as isotonic Lugol’s solutions (Fig. 2). Furthermore, we observed that the pH decreased significantly over time and regression analysis showed that decreasing pH—and not tonicity of the solution—is the most significant parameter associated with tissue shrinkage.

As pH and tissue shrinkage turned out to be strongly related, we sought to stabilize the pH in order to diminish soft-tissue shrinkage artefacts. Results from the experiments with mouse livers and human fetuses showed that staining in B-Lugol resulted in pH stabilization of the solution during the entire staining period (Figs. 4C and 5). By keeping the pH of the staining solution constant, tissue shrinkage of ex vivo mice livers and of entire fetuses was hardly observed during the entire staining period (Figs. 4A and 5). However, the volume of the body and kidneys of fetus #1 (Fig. 5A) and lungs of fetus #2 (Fig. 5B), were affected, showing a decrease of 8%. We speculate that this difference in shrinkage could possibly be due to the fact that fetus #1 was two weeks younger; having a less mineralized skeleton and as such being more prone to volume changes. Further research using more fetuses of different developmental ages should address the possibility of differential shrinkage between organs across different gestational ages. Furthermore, we did not observe any negative effects of buffering Lugol’s solution with Sorenson’s buffer, because triiodide uptake and staining time remained similar compared to Lugol. However, the triiodide concentration of B-Lugol dropped slightly more than of Lugol (Fig. 4D), this is possibly due to the lack of shrinkage of the mouse livers stained in B-Lugol. Hence, these livers have a relatively larger volume, therefore more tissue volume is available for the uptake of triiodide.

Although Lugol gradually becomes acidic on the shelf (approximately pH 4, Supplemental Fig. 2), this acidification does not explain the observed exponential decrease in pH levels during tissue staining (Fig. 2C). Because of the log-based pH, one could understand that the H^+^-ion concentration increases linearly with the logarithm of staining time. A possible explanation for this initially quick decrease in pH might be due to the release of free excess formaldehyde (the monomeric product of PFA) from the tissue into the solution; formaldehyde is subsequently degraded into formic acid^16^. Furthermore, such release of free formaldehyde into the staining solution might also explain the small increase in osmolarity that was observed; freshly dissolved 4% PFA in PBS has a high osmolarity (1607 mOsm). This idea is underscored by the observation that adding freshly dissolved 4% PFA solution to isotonic 3.75% Lugol’s solution enhances the acidification process significantly and is proportional to the amount of formaldehyde added (Supplemental Fig. 2). Moreover, in immunohistochemistry the cross-linking of proteins in formaldehyde-fixed tissue can be reversed using antigen unmasking solutions^17^. Such antigen unmasking solutions are most often acidic citrate based solutions^18^ which, in combination with high temperature, will quickly release the covalent linked formaldehyde from the fixed tissue. We propose that the tendency of Lugol’s solution to acidify over time, in combination with the long staining time, releases formaldehyde bound to the tissue which subsequently promotes further acidification of the staining solution and enhances this effect. Therefore, we tested, as is common practice in histological procedures, various washing steps prior to the start of the staining procedure, to remove excess free formaldehyde and impregnate tissue with buffer. However, this procedure did not improve the stabilisation of pH, nor did it prevent tissue shrinkage (data not shown).

Even though this study provides strong evidence that pH is a key factor in soft-tissue shrinkage, the molecular mechanisms between acidification and tissue shrinkage remain unclear. Nevertheless, one could speculate that during staining of the tissue, i.e. uptake of I_3_^-^ in the tissue, a surplus of K^+^ is created in the staining solution. These K^+^ ions could hold negative hydroxide molecules, allowing free hydrogen atoms to accumulate in the solution, thus lowering the pH. Further research will be required to improve the washing protocol and the buffering of the triiodide solution and thus provide answers on the mechanisms underlying the observed pH-dependent shrinkage. Nevertheless, our newly developed and tested buffered hypertonic 3.75% B-Lugol already shows that B-Lugol can serve as a practical alternative for Lugol because it provides pH stability, thus reducing tissue shrinkage and deformation without affecting the time required to reach sufficient intensity of staining.

In conclusion, we provide evidence that the acidification of Lugol’s solution is the key factor in soft-tissue shrinkage rather than the osmolarity of the staining solution. We showed that staining in Lugol’s solution prepared in Sorensen’s buffer (B-Lugol) leads to a stable pH and almost completely prevents soft-tissue shrinkage, without affecting the staining process or timing.

## Methods

### Mouse livers

Mouse livers of the FVB (Friend leukemia Virus B) strain were used as model tissue, because of the homogeneity of the tissue and the size of the organ, allowing the preparation of multiple highly similar samples. In total 39 adult mouse livers were collected and fixed for 48 h in freshly dissolved 4% paraformaldehyde (PFA) (w/v) in Phosphate Buffered Saline (PBS, 10 mM H_2_NaPO_4_/HNa_2_PO_4_, 150 mM NaCl, pH 7.4) at room temperature. These FVB mice were not bred for the purpose of this study, but were surplus mice of breeding or of other research projects. After fixation the livers were stored in a storage solution (0.2% PFA in PBS) at room temperature for 48 h up until staining.

### Human fetuses

Three human fetuses, donated to the Dutch Fetal Biobank after termination of pregnancy. After a written maternal informed consent, the donated fetuses were completely anonymized. Research has been approved by the Medical Ethical Committee (MEC) and Biobank Committee (BC) of the Amsterdam University Medical Centers, location AMC, Amsterdam, the Netherlands (METC 2016_285, #B2017369). Fetus #1: 13 + 1 weeks of gestation (weeks + days), total length = 8 cm, weight = 17 g. Fetus #2: 15 + 2 weeks of gestation, total length = 15 cm, weight = 58 g. Fetus #3: 16 + 2 weeks of gestation, total length = 17 cm, weight = 85 g. After acquisition, the fetuses were fixed in 4% PFA in PBS for 4 days at 4 °C and subsequently stored in storage solution (0.2% PFA in PBS) at 4 °C.

### Different Lugol solutions

To study the relations between tissue shrinkage and tonicity and/or acidification of Lugol, five different Lugol’s solutions containing different I_2_KI concentrations and tonicities were prepared from a 15% stock solution of Lugol (10 g KI and 5 g I_2_ dissolved in 100 mL bi-distilled water). From this stock solution isotonic 3.75%, hypotonic 2.5% and hypotonic 1.25% Lugol were prepared by dilution in bi-distilled water. To prepare isotonic solutions with 2.5% and 1.25% of Lugol, the 3.75% isotonic solution was further diluted in PBS. 3.75% buffered Lugol (B-Lugol) was prepared by combining in an 1:1 ratio 7.5% Lugol’s solution with 2 × Sorensen’s buffer (71.5 mL 266 mM Na_2_HPO_4_ and 28.5 mL 266 mM KH_2_PO_4_ to pH 7.2) (Supplementary PDF: formulas and protocols). 7.5% Lugol was prepared by diluting the 15% stock solution with bi-distilled water. As a control, in the comparison of the effects of different Lugol staining solutions, three fixed livers were kept in storage solution.

### Staining and evaluation procedure

Specimens were stained in a volume of Lugol’s solution equivalent of 20 times their weight for the mouse livers and 10 times their weight for the fetal specimens at room temperature. Before and during the experiments, pH, osmolarity and optical density (OD) of the staining solutions were measured at predefined time points. pH was measured using a Consort P901 Electrochemical analyzer (Consort bvba, Turnhout, Belgium). Osmolarity was measured using a Gonotec Osmomat 030 cryoscopic osmometer (Gonotec, Berlin, Germany). OD was measured spectrophotometrically using a Nanodrop ND-1000 (Thermo Fisher Scientific, United States, Thermo Fisher | AMIRA^19^) with function UV–Vis at 550 nm. To convert the observed OD value in the Lugol solutions at the different time points into the concentration of triiodide in the solution a calibration curve of OD versus defined Lugol concentrations was prepared by serial dilution of the Lugol’s stock solution (Supplementary Fig. 1); assuming that the decrease of triiodide concentration in the staining solution reflects the uptake of triiodide in the tissue.

### Visualization of the Lugol staining

The mouse livers were scanned using a SOMATOM clinical CT scanner (Siemens Healthineers AG, Erlangen, Germany). Scans were made before the start of the staining and subsequently every two hours up to 8 h of staining. Thereafter, the samples were scanned depending on scanner availability. All livers were scanned in one scan session, using the following settings: X-ray tube voltage = 150 kV, X-ray tube current = 200 mAs, voxel size = 0.2 × 0.2 × 0.1 mm anisotropic and scan time depended on the chosen field of view (30–60 s). The DICOM-files were imported in software package AMIRA (version 2019.3, Thermo Fisher Scientific, USA, Thermo Fisher | AMIRA^19^). This package was used to determine liver volumes and to evaluate staining. Liver volumes were determined by automatically segmenting the 2D-images using specific threshold grey values which were adapted depending on the intensity of staining. Volume was then calculated as segmented tissue area times slice thickness. Staining was considered complete when the center of the liver reached the maximum detectable value (around 3000 Hounsfield Units).

The three human fetuses were scanned using magnetic resonance imaging to determine total body and organ volumes prior to staining with B-Lugol. The largest fetus was scanned using the clinical 3 T MRI scanner (Philips Healthcare, Best, The Netherlands) with a bore diameter of 70 cm and integrated gradient coils, producing a maximum amplitude of 45 mT/m. A 160 mm knee coil was used for radio frequency excitation and signal reception. T1 weighted images were produced with the following parameters: echo time (ET) = 4.4 ms, repetition time (RT) = 18 ms, number of signal averages (NSA) = 3, total scanning time = 50 min and voxel size = 0.33 mm isotropic. The two smaller fetuses were scanned using the preclinical 7 T MRI scanner (MR Solutions, Guildford, UK) with a bore diameter of 17 cm and a 70 mm rat body coil, reaching a maximum magnetic field strength of 600 mT/m. T1 weighted images were produced with the following parameters: ET = 7 ms, RT = 20 ms, NSA = 16, total scanning time = 3 h 17 min–5 h 50 min (depending on fetal size) and voxel size = 0.156–0.195 mm isotropic (depending on fetal size). The fetuses were embedded in alginate to stabilize them during MRI scanning. During Lugol’s staining the fetuses were regularly scanned using the clinical CT scanner to evaluate the progress of the staining. Scanner settings were: X-ray tube voltage = 150 kV, X-ray tube current = 200 mAs, voxel size = 0.1 mm isotropic and scan time depended on fetal size. Staining was considered complete when the vessels in the center of liver were completely visible. The fetuses were then scanned using a GE Phoenix v|tome|x m tomographer (General Electric, Wunstorf, Germany). The voltage (180–210 kV) and current (180–210 µA) were adjusted to the fetal size. A full scan consisted of 1500 projections that were made with continuous sample rotation over 360°. One saved projection is the average of 4 images, where every image is acquired with an exposure time of 333 ms, giving an acquisition time of 33 min per scan. To avoid beam hardening, a 0.5 mm copper (Cu) filter was applied. All fetuses were scanned in two parts, first the cranial part and then the caudal part with overlap, to enable a shorter source-to-object distance, resulting in a higher spatial resolution (voxel size = 20–50 µm)^7^. Phoenix datos|x (version 2.2, Baker Hughes, Texas, USA, Baker Huges | Phoenix^20^) software was used to reconstruct the raw scan data.

The MRI and CT images were converted into the DICOM format and imported into AMIRA (version 2019.3, Thermo Fisher Scientific, USA, Thermo Fisher | AMIRA^19^) software. Total body and different organs (liver, lung, kidney and brain) were manually segmented in 2D-images using a systematic random sample of one in three, five or ten slices, with the first slice picked randomly in this range, to determine the total volume of each structure using AMIRA^21,22^. Body and organ volumes were calculated as the sum of the segmented tissue area times slice distance. Unfortunately, due to damage to the head, reliable brain segmentation and volume estimation of the brain was not possible for fetus #3.

### Statistical analysis

Because initial volumes of the livers differed, the observed liver volumes were corrected by determining and applying a correction factor per liver^15^ and normalized by setting the mean volume at time 0 to 100% (Table 1). Statistical analyses were performed using SPSS (version 24, IBM, Chicago, USA, IBM | SPSS^23^). To assess the correlation between tissue volume, osmolarity and pH without the confounding effect of time, a Pearson’s partial correlation test, controlling for time, was performed. Because of the correlation result, and literature data suggesting an effect of osmolarity, a stepwise multiple regression analysis, consisting of linear regression of tissue volume on pH followed by regression of the residual volume on osmolarity, was performed. We did not include the controls in the regression analysis because the tissue volume, acidity and osmolarity did not show any changes. A *p*-value equal or below 0.05 was considered to indicate statistical significance. The results of these analysis are presented in Tables 2A and 2B for the first and second experiment with mouse livers, respectively.

### Method statement

All methods were carried out in accordance with relevant guidelines and regulations. The study was carried out in accordance to the ARRIVE guideline^24^.

## Supplementary Information

Supplementary Information.
