# Author Correction: Reducing soft-tissue shrinkage artefacts caused by staining with Lugol’s solution

**DOI:** 10.1038/s41598-022-06160-4

**Published:** 2022-02-07

**Authors:** Y. Dawood, J. Hagoort, B. A. Siadari, J. M. Ruijter, Q. D. Gunst, N. H. J. Lobe, G. J. Strijkers, B. S. de Bakker, M. J. B. van den Hoff

**Affiliations:** 1 Obstetrics and Gynaecology, Amsterdam Reproduction and Development Research Institute, Amsterdam UMC, University of Amsterdam, Meibergdreef 9, Amsterdam, The Netherlands; 2 Medical Biology, Amsterdam UMC, University of Amsterdam, Meibergdreef 15, Amsterdam, The Netherlands; 3 Biomedical Engineering and Physics, Amsterdam UMC, University of Amsterdam, Meibergdreef 9, Amsterdam, The Netherlands; 4 Radiology, Amsterdam UMC, University of Amsterdam, Meibergdreef 9, Amsterdam, The Netherlands

Correction to: *Scientific Reports* 10.1038/s41598-021-99202-2, published online 05 October 2021

The Supplementary Information file published with this Article contained an error in the amounts used to prepare the Sorensens buffer, under the subheading ‘2. Buffered Lugol’s solution (B-Lugol)’.

“Before preparing the buffered Lugol’s solution, prepare a two times Sörensen’s buffer (pH 7.2). See below for the appropriate amounts and ratio.

**Table:** 

266 mM Na_2_HPO_4_	71.52 gram Na_2_HPO_4_.5H_2_0 in 1L bi-distilled water
266 mM KH_2_PO_4_	18.16 gram KH_2_PO_4_ in 1L bi-distilled water

Combine 71.5 ml Na_2_HPO_4_ with 28.5 ml KH_2_PO_4_ for a pH of 7.2.”

now reads:

“Before preparing the buffered Lugol’s solution, prepare a 266 mM Sorensen’s buffer (pH 7.2). See below for the appropriate amounts and ratio.

**Table:** 

266 mM Na_2_HPO_4_	47.35 gram Na_2_HPO_4_.2H_2_0 in 1L bi-distilled water
266 mM KH_2_PO_4_	36.20 gram KH_2_PO_4_ in 1L bi-distilled water

Combine 71.5 ml Na_2_HPO_4_ with 28.5 ml KH_2_PO_4_ for a pH of 7.2.”

This error has now been corrected in the Supplementary Information file that accompanies the original Article.

