# Supplementary material for: Reducing soft-tissue shrinkage artefacts caused by staining with Lugol’s solution
Source: Sci Rep. 2021 Oct 5;11:19781. doi: 10.1038/s41598-021-99202-2 (PMC8492742; doi:10.1038/s41598-021-99202-2)
Supplement: Supplementary file 1 — Supplementary Information. [file 41598_2021_99202_MOESM1_ESM.pdf]

Supplementary material

Reducing soft-tissue shrinkage artefacts caused by staining with Lugol's solution.

Y. Dawood,[1,2] J. Hagoort,[2] B.A. Siadari,[2] J.M. Ruijter,[2] Q.D. Gunst,[2] N.H.J. Lobe,[4] G.J. Strijkers,[3] B.S. de Bakker,[2] M.J.B. van den Hoff[2]

[1]Amsterdam UMC, University of Amsterdam, Obstetrics and Gynaecology, Amsterdam Reproduction & Development Research Institute, Meibergdreef 9, Amsterdam, The Netherlands

[2]Amsterdam UMC, University of Amsterdam, Medical Biology, Meibergdreef 15, Amsterdam, The Netherlands

[3]Amsterdam UMC, University of Amsterdam, Biomedical Engineering and Physics, Meibergdreef 9, Amsterdam, The Netherlands

[4]Amsterdam UMC, University of Amsterdam, Radiology, Meibergdreef 9, Amsterdam, The Netherlands

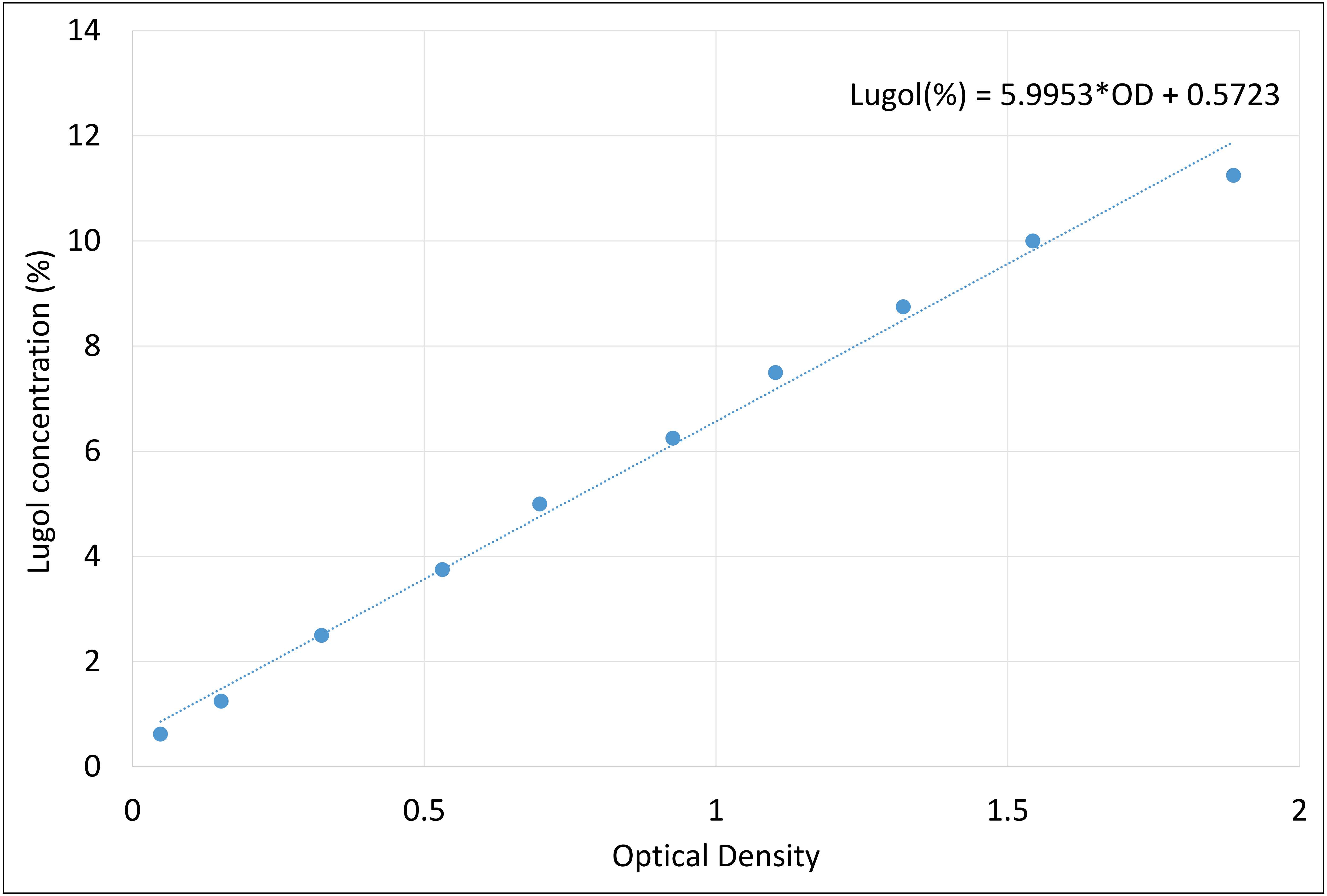

Supplemental Figure 1. Calibration curve of optical density to Lugol concentration. OD was measured spectrophotometrically using a Nanodrop ND-1000 (Thermo Fisher Scientific, United States) with function UV-Vis at 550 nm. The calibration curve was made by measuring the OD value of known amounts of I2KI solutions by serial dilution of Lugol stock solution. The relation of is given by the following formula: Lugol(%) = 5.9953*OD + 0.5723, in which Lugol(%) is the concentration of Lugol in the solution and OD is the optical density measured at 550 nm.

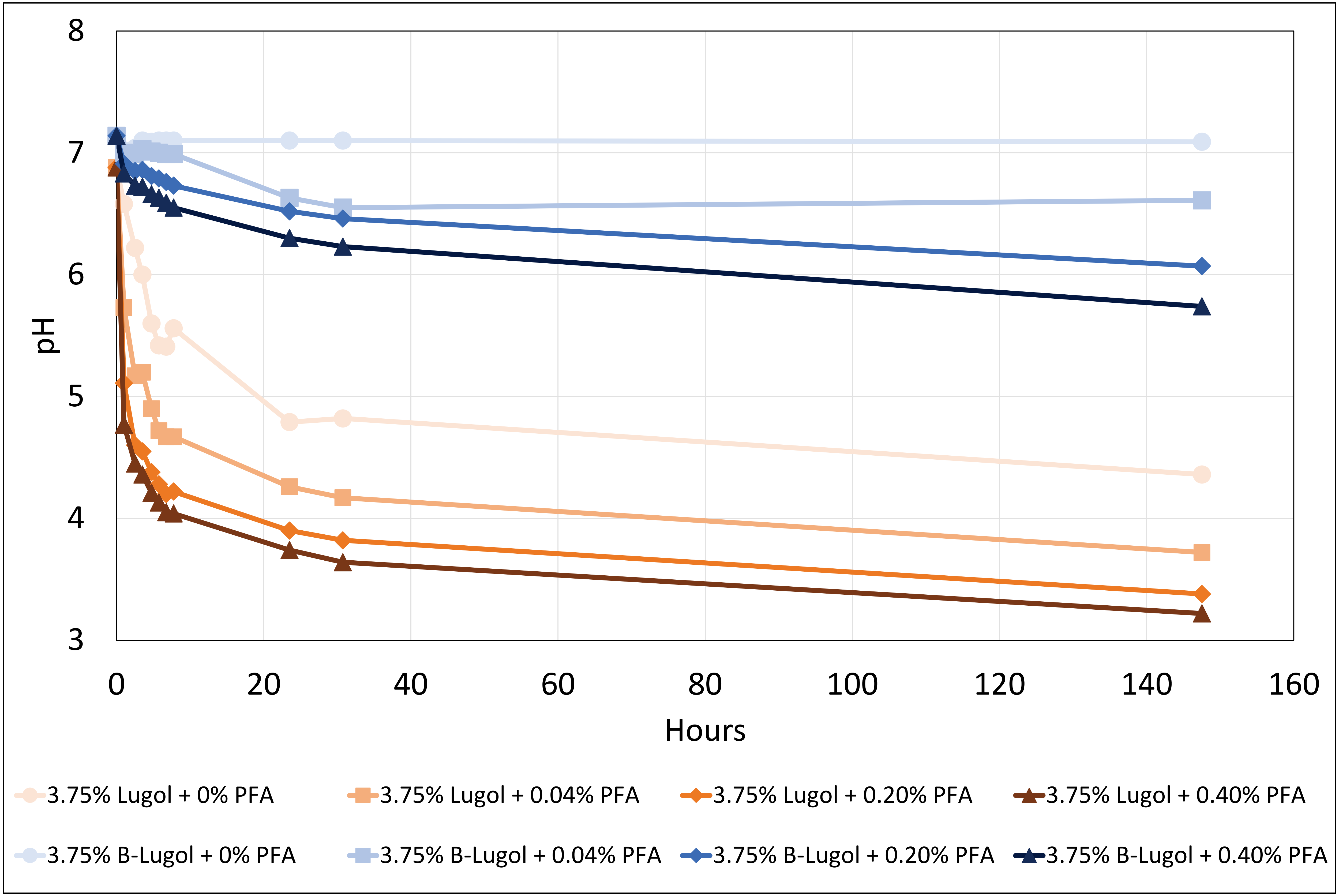

Supplemental Figure 2. Effect of addition of paraformaldehyde (PFA) to Lugol's solution (Lugol) and buffered Lugol's solution (B-Lugol). Eppendorf tubes (2 ml) were filled with 1.5 ml of 3.75% Lugol or 3.75% B-Lugol and supplemented without or with different amounts of PFA in 0.5 ml PBS as indicated in the legend of the graph. Without addition of PFA the Lugol becomes gradually acidic (pH = 4.4 after 147 hours), while the pH of B-Lugol remains stable (pH = 7.1 after 147 hours). With addition of PFA, pH decreases in both Lugol and B-Lugol, though to a much higher extent in Lugol (pH = 3.2 - 3.7) compared to B-Lugol (pH = 5.7 - 6.6). This effect is larger with the addition of more PFA.

Supplemental PDF: formulas and protocols

1. **Lugol's solution**

The success of staining is mainly dependent on three factors: 1) specimen size, 2) staining solution concentration and 3) staining time. In larger specimens the staining fluid has to penetrate deeper to reach the core of the sample. Staining concentration and time are interdependent factors. A higher concentration results in faster diffusion of the staining solution, enabling shorter staining exposure times. However, extended exposure time (to ensure complete and even staining) with higher concentration can result in overstaining and loss of tissue differentiation and/or tissue shrinkage. See table 1 for the appropriate amounts of solvent and solutes needed to mix the appropriate Lugol (potassium triiodide; I_2K) concentration. To vary in tonicity PBS can be used as solvent next to bi-distilled water.

Target Lugol w/v (%)	Tonicity	I2 (grams)*	KI (grams)*	Volume of PBS (ml)	End volume with bi-distilled water (ml)
1.25	Hypotonic	0.42	0.83	0	100
1.25	Isotonic	0.42	0.83	67	100
2.5	Hypotonic	0.84	1.67	0	100
2.5	Isotonic	0.84	1.67	33	100
3.75	Isotonic	1.25	2.50	0	100
5.0	Hypertonic	1.67	3.33	0	100
7.5	Hypertonic	3.50	7.50	0	100
15	Hypertonic	5	10	0	100

Table 1. Lugol solutes and solvent ratios. To vary in tonicity PBS can be used with bi-distilled water to reach the desired osmolarity. *We use I2 and KI 99+% from Fischer Scientific.

Grind the solid I_2 using a mortar and pestle for quicker dissolvent. Put the I_2 and KI (and PBS) in a glass Erlenmeyer and bring to an end volume of 100 ml with bi-distilled water. For convenience use magnetic stirrer. The powder should be dissolved in a couple of minutes, depending on the concentration and quantity of the solution. Always work under a fume hood and store the Lugol's solution in the dark.

2. Buffered Lugol's solution (B-Lugol)

Before preparing the buffered Lugol's solution, prepare a 266mM Sorensen's buffer (pH 7.2). See below for the appropriate amounts and ratio.

266 mM Na_2HPO_4	47.35 gram $Na_2HPO_4.2H_20$ in 1L bi-distilled water
266 mM KH_2PO_4	36.20 gram KH_2PO_4 in 1L bi-distilled water

Combine 71.5 ml Na_2HPO_4 with 28.5 ml KH_2PO_4 for a pH of 7.2.

Preparation of B-Lugol

The easiest way is to prepare a 15% Lugol's solution (see table 1) and combine and dilute appropriately (table 2).

Target Lugol w/v (%)	Volume of 15% Lugol stock (ml)	Volume of bi-distilled water (ml)	Volume of Sorensen's buffer (ml)	Total solution volume (ml)
1.25	8.33	41.67	50	100
2.5	16.67	33.33	50	100
3.75	25	25	50	100

Table 2. B-Lugol: Lugol and Sorensens buffer ratios. Lugol stock was made by dissolving 5 gram I_2 with 10 gram KI in 100 ml bi-distilled water. Sorensen's buffer = 266 mM, pH 7.2.

3. Staining with B-Lugol

Rinse the samples prior to staining with B-Lugol using PBS. After washing, take the samples out and immerse in B-Lugol. See table 2 for preparation of B-Lugol. We use a 3.75% B-Lugol; however depending on the size of the sample, lower concentrations can also be used. **NOTE:** Put the sample in an amount of volume at least equal to 20 times the weight of the sample. The higher the amount of volume of B-Lugol, the more stable the pH will remain.

Staining time depends on the size of the sample and the concentration of B-Lugol used. If you have ready access to a medical or micro-CT scanner, running shorter, low-quality scans iteratively is useful for checking the progress of staining before investing more time and money into longer, high-quality scans.

During staining measure the pH level, osmolarity and optical density (OD). We would advise to replace the solution with new B-Lugol if the pH drops below 6.8

Experiment 1

Mouse livers were stained in Lugol's solution with different Lugol concentration and osmolarity. The contention is that tissue shrinkage is dependent on osmolarity. The graphs of volume and osmolarity against time show this shrinkage over time associated with an increase in osmolarity over time. However, the graph of volume against osmolarity shows that the whole volume range is present in each solution, despite the large difference in osmolarity. This pleads against osmolarity as major factor in tissue shrinkage.

Graph

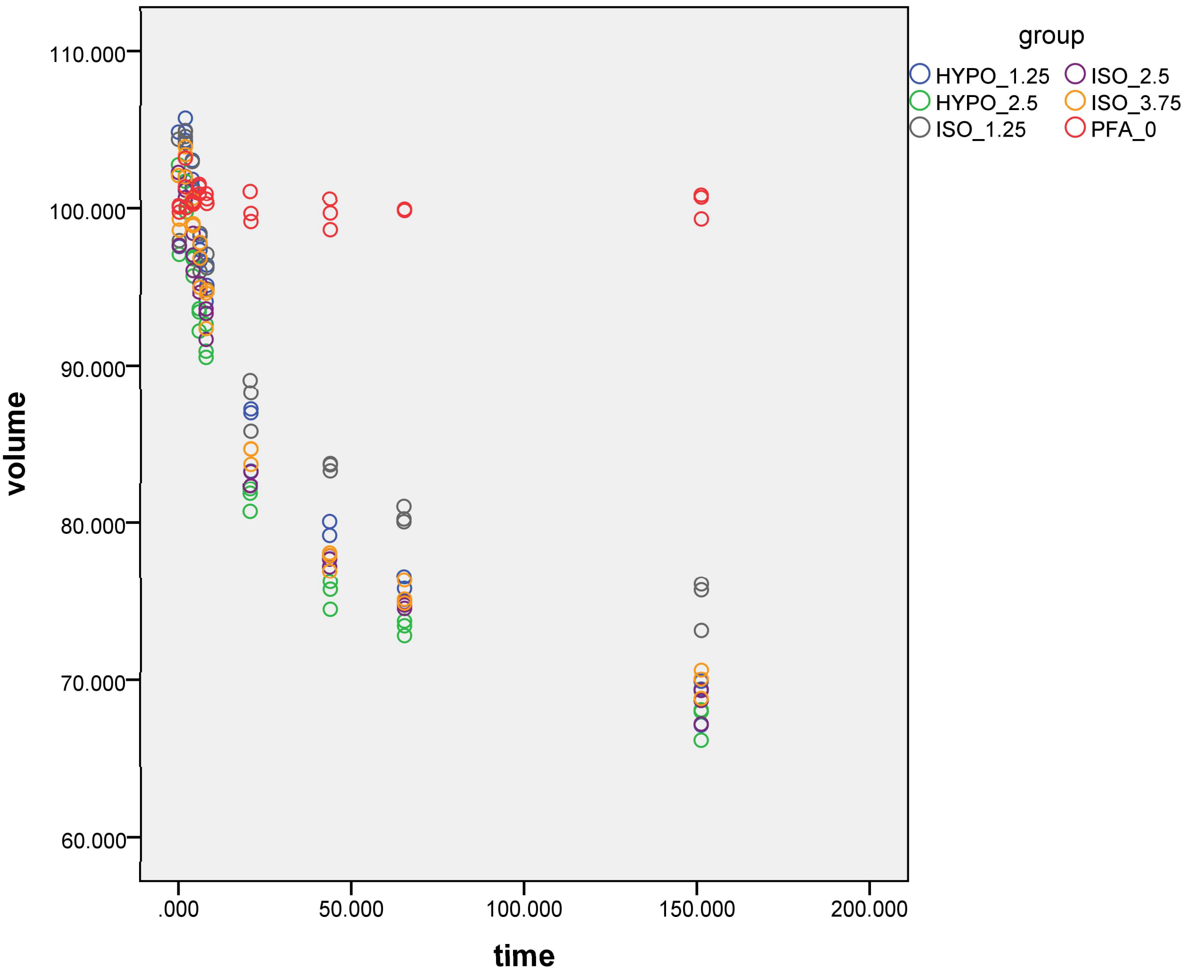

Graph

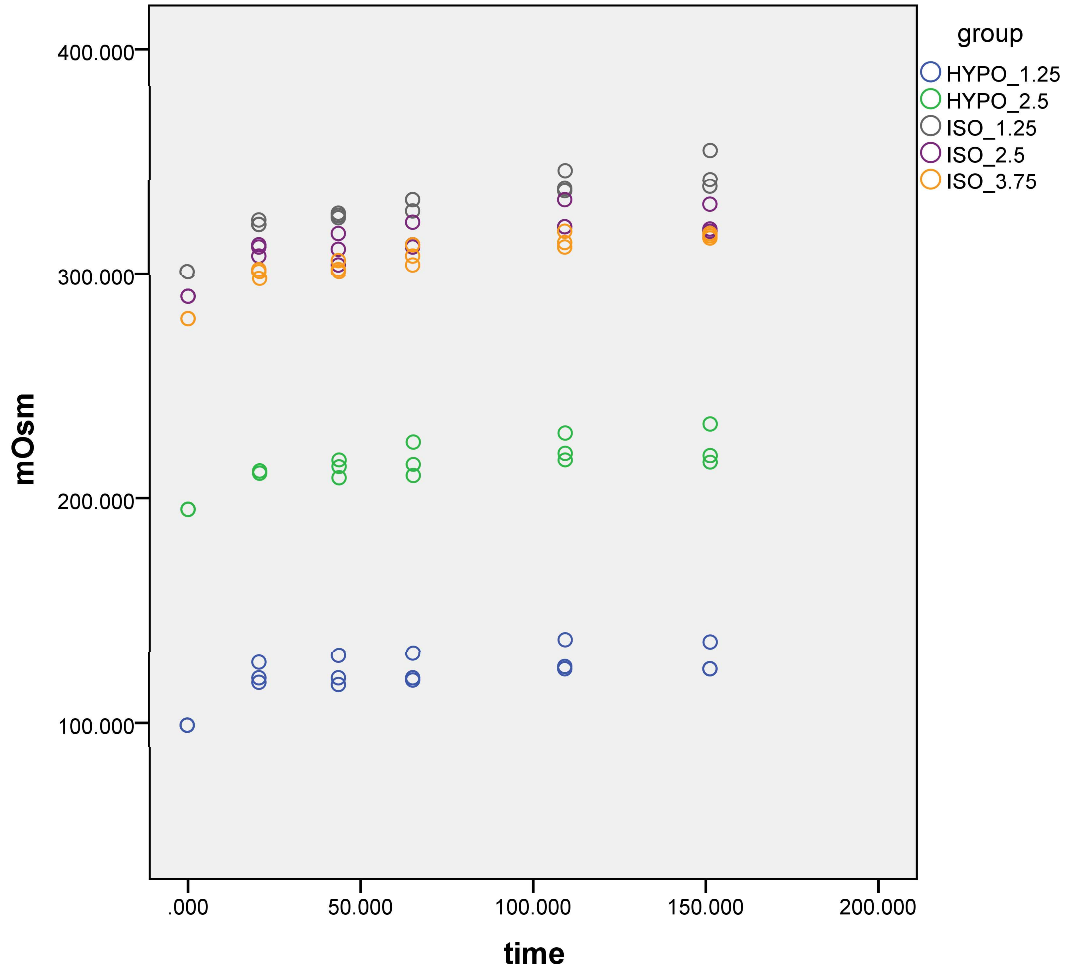
group
HYPO_1.25
HYPO_2.5
ISO_1.25
ISO_2.5
ISO_3.75
400.000
300.000
200.000
100.000
mOsm
.000
50.000
100.000
150.000
200.000
time

Graph

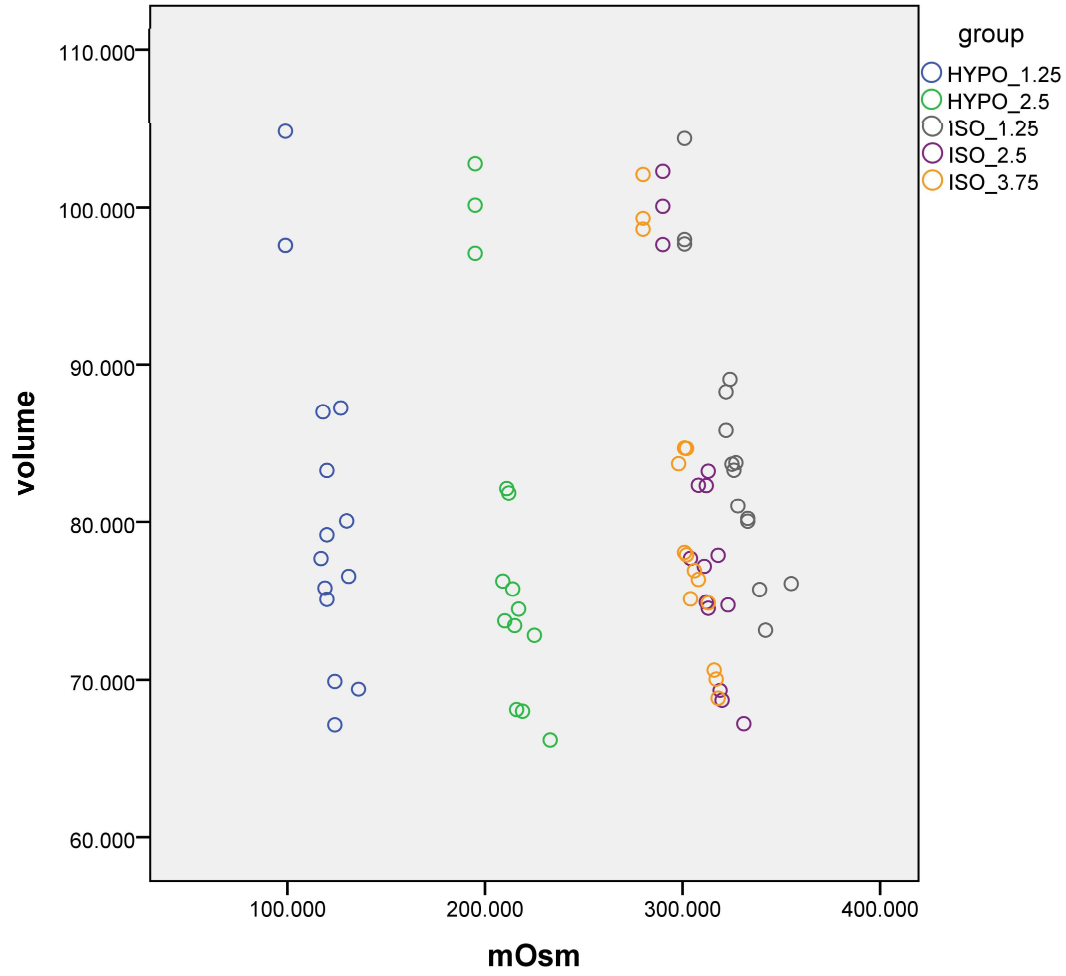
group
HYPO_1.25
HYPO_2.5
ISO_1.25
ISO_2.5
ISO_3.75
110.000
100.000
90.000
80.000
70.000
60.000
volume
100.000
200.000
300.000
400.000
mOsm

Correlations

Pearson correlation shows that most variables are correlated to each other. However, this is because they are all, except osmolarity, correlated with time. Partial correlation analysis, controlling for the effect of time, shows that only pH is correlated significantly with tissue volume.

Correlations

		time	mOsm	OD	pH	volume
time	Pearson Correlation	1	.119	-.385**	-.734**	-.852**
	Sig. (2-tailed)		.266	.000	.000	.000
	N	150	89	135	135	135
mOsm	Pearson Correlation	.119	1	.124	-.003	-.075
	Sig. (2-tailed)	.266		.294	.980	.523
	N	89	89	74	74	74
OD	Pearson Correlation	-.385**	.124	1	.241**	.428**
	Sig. (2-tailed)	.000	.294		.005	.000
	N	135	74	135	135	135
pH	Pearson Correlation	-.734**	-.003	.241**	1	.848**
	Sig. (2-tailed)	.000	.980	.005		.000
	N	135	74	135	135	135
volume	Pearson Correlation	-.852**	-.075	.428**	.848**	1
	Sig. (2-tailed)	.000	.523	.000	.000	
	N	135	74	135	135	135

**. Correlation is significant at the 0.01 level (2-tailed).

Partial Corr

Correlations

Control Variables			mOsm	OD	pH	volume
time	mOsm	Correlation	1.000	.185	.125	.050
		Significance (2-tailed)	.	.117	.291	.674
		df	0	71	71	71
	OD	Correlation	.185	1.000	-.066	.208
		Significance (2-tailed)	.117	.	.446	.016
		df	71	0	132	132
	pH	Correlation	.125	-.066	1.000	.627
		Significance (2-tailed)	.291	.446	.	.000
		df	71	132	0	132
	volume	Correlation	.050	.208	.627	1.000
		Significance (2-tailed)	.674	.016	.000	.
		df	71	132	132	0

Graph

This graph illustrates the relation between volume and pH (note that with time the volume decreases

with dreasing pH, to the left-bottom corner of the graph) in each of the solutions

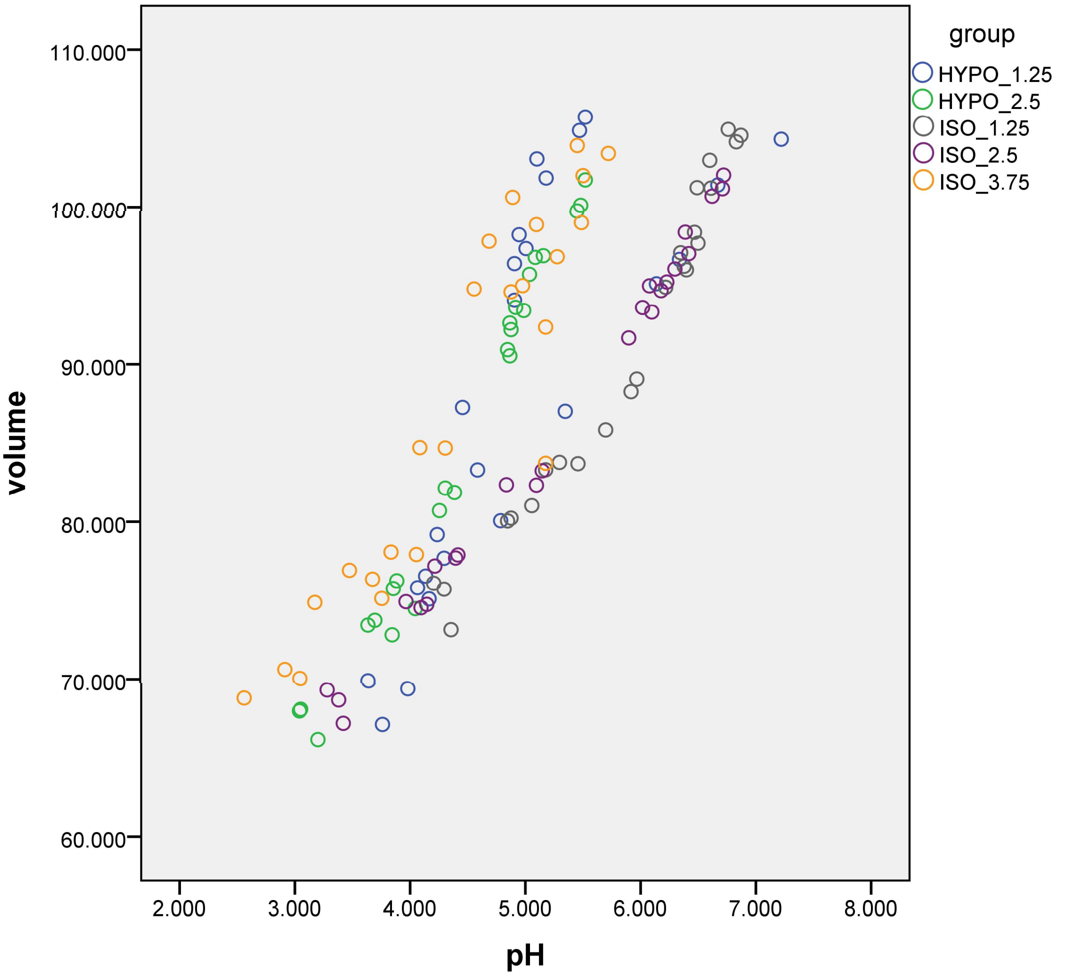

To describe this linear relation between tissue volume and pH for each group a regression analysis of volume on pH was performed, saving the predicted values and the residuals for further analysis.

Regression

Model Summary[b]

group	Model	R	R Square	Adjusted R Square	Std. Error of the Estimate
HYPO_1.25	1	.804[a]	.646	.630	7.715737
HYPO_2.5	1	.986[a]	.972	.970	1.994157
ISO_1.25	1	.977[a]	.954	.952	2.245579
ISO_2.5	1	.995[a]	.990	.990	1.154819
ISO_3.75	1	.930[a]	.864	.858	4.471965

a. Predictors: (Constant), pH

b. Dependent Variable: volume

ANOVA[a]

group	Model		Sum of Squares	df	Mean Square	F	Sig.
HYPO_1.25	1	Regression	2394.886	1	2394.886	40.228	.000[b]
		Residual	1309.717	22	59.533		
		Total	3704.603	23			
HYPO_2.5	1	Regression	3002.026	1	3002.026	754.911	.000[b]
		Residual	87.487	22	3.977		
		Total	3089.513	23			
ISO_1.25	1	Regression	2308.838	1	2308.838	457.864	.000[b]
		Residual	110.938	22	5.043		
		Total	2419.776	23			
ISO_2.5	1	Regression	2992.579	1	2992.579	2243.974	.000[b]
		Residual	29.339	22	1.334		
		Total	3021.918	23			
ISO_3.75	1	Regression	2802.413	1	2802.413	140.131	.000[b]
		Residual	439.966	22	19.998		
		Total	3242.380	23			

a. Dependent Variable: volume

b. Predictors: (Constant), pH

Coefficients[a]

			Unstandardized Coefficients		Standardized Coefficients			95.0% Confidence ...
group	Model		B	Std. Error	Beta	t	Sig.	Lower Bound
HYPO_1.25	1	(Constant)	34.057	8.749		3.893	.001	15.912
		pH	11.017	1.737	.804	6.343	.000	7.415
HYPO_2.5	1	(Constant)	19.161	2.427		7.894	.000	14.128
		pH	14.835	.540	.986	27.476	.000	13.716
ISO_1.25	1	(Constant)	22.906	3.207		7.143	.000	16.256
		pH	11.669	.545	.977	21.398	.000	10.538
ISO_2.5	1	(Constant)	35.208	1.102		31.946	.000	32.923
		pH	9.707	.205	.995	47.371	.000	9.282
ISO_3.75	1	(Constant)	35.130	4.521		7.770	.000	25.754
		pH	11.887	1.004	.930	11.838	.000	9.804

Coefficients[a]

group	Model		95.0% Confidence ... Upper Bound
HYPO_1.25	1	(Constant)	52.202
		pH	14.620
HYPO_2.5	1	(Constant)	24.195
		pH	15.955
ISO_1.25	1	(Constant)	29.556
		pH	12.800
ISO_2.5	1	(Constant)	37.494
		pH	10.131
ISO_3.75	1	(Constant)	44.506
		pH	13.969

a. Dependent Variable: volume

This regression analysis showed that the intercept and slope parameters (values on the line (constant) and pH, respectively in the above table) have overlapping 95% confidence intervals. This shows that the relation between pH and tissue volume follows similar 'rules' in each of the staining solutions.

To further dissect the role of osmolarity a regression analysis of the residual volume on Osmolarity was performed.

Regression

Model Summary[b]

group	Model	R	R Square	Adjusted R Square	Std. Error of the Estimate
HYPO_1.25	1	.225[a]	.051	-.044	3.52726922
HYPO_2.5	1	.043[a]	.002	-.109	2.72415041
ISO_1.25	1	.863[a]	.745	.720	1.31037099
ISO_2.5	1	.066[a]	.004	-.095	1.39834820
ISO_3.75	1	.650[a]	.422	.364	3.45849238

a. Predictors: (Constant), mOsm

b. Dependent Variable: Unstandardized Residual

ANOVA[a]

group	Model		Sum of Squares	df	Mean Square	F	Sig.
HYPO_1.25	1	Regression	6.660	1	6.660	.535	.481[b]
		Residual	124.416	10	12.442		
		Total	131.076	11			
HYPO_2.5	1	Regression	.125	1	.125	.017	.900[b]
		Residual	66.789	9	7.421		
		Total	66.914	10			
ISO_1.25	1	Regression	50.219	1	50.219	29.247	.000[b]
		Residual	17.171	10	1.717		
		Total	67.390	11			
ISO_2.5	1	Regression	.086	1	.086	.044	.839[b]
		Residual	19.554	10	1.955		
		Total	19.639	11			
ISO_3.75	1	Regression	87.280	1	87.280	7.297	.022[b]
		Residual	119.612	10	11.961		
		Total	206.892	11			

a. Dependent Variable: Unstandardized Residual

b. Predictors: (Constant), mOsm

Coefficients[a]

			Unstandardized Coefficients		Standardized Coefficients			95.0% Confidence ...
group	Model		B	Std. Error	Beta	t	Sig.	Lower Bound
HYPO_1.25	1	(Constant)	12.099	21.983		.550	.594	-36.881
		mOsm	-.130	.177	-.225	-.732	.481	-.525
HYPO_2.5	1	(Constant)	2.852	26.156		.109	.916	-56.317
		mOsm	-.016	.121	-.043	-.130	.900	-.289
ISO_1.25	1	(Constant)	-72.687	13.312		-5.460	.000	-102.347
		mOsm	.217	.040	.863	5.408	.000	.128
ISO_2.5	1	(Constant)	3.671	18.303		.201	.845	-37.111
		mOsm	-.012	.058	-.066	-.209	.839	-.141
ISO_3.75	1	(Constant)	-124.181	45.241		-2.745	.021	-224.983
		mOsm	.398	.147	.650	2.701	.022	.070

Coefficients[a]

group	Model		95.0% Confidence ... Upper Bound
HYPO_1.25	1	(Constant)	61.079
		mOsm	.265
HYPO_2.5	1	(Constant)	62.021
		mOsm	.258
ISO_1.25	1	(Constant)	-43.027
		mOsm	.307
ISO_2.5	1	(Constant)	44.453
		mOsm	.117
ISO_3.75	1	(Constant)	-23.379
		mOsm	.726

a. Dependent Variable: Unstandardized Residual

This regression analysis showed that in most solutions the tissue volume could not be further explained by a role for osmolarity. The intercept and slope parameters were not, or hardly, different from 0.

Experiment 2

Mouse livers were stained in conventional Lugol's solution (Lugol) and buffered Lugol's solution (B-Lugol). The first experiment (see above) showed that tissue shrinkage could be attributed to the decreasing pH over staining time. To counteract this shrinkage during staining, a strong buffer was used to prevents acidification of the staining solution. The graph of volume against time shows indeed that in B-Lugol most tissue shrinkage is prevented.

Graph

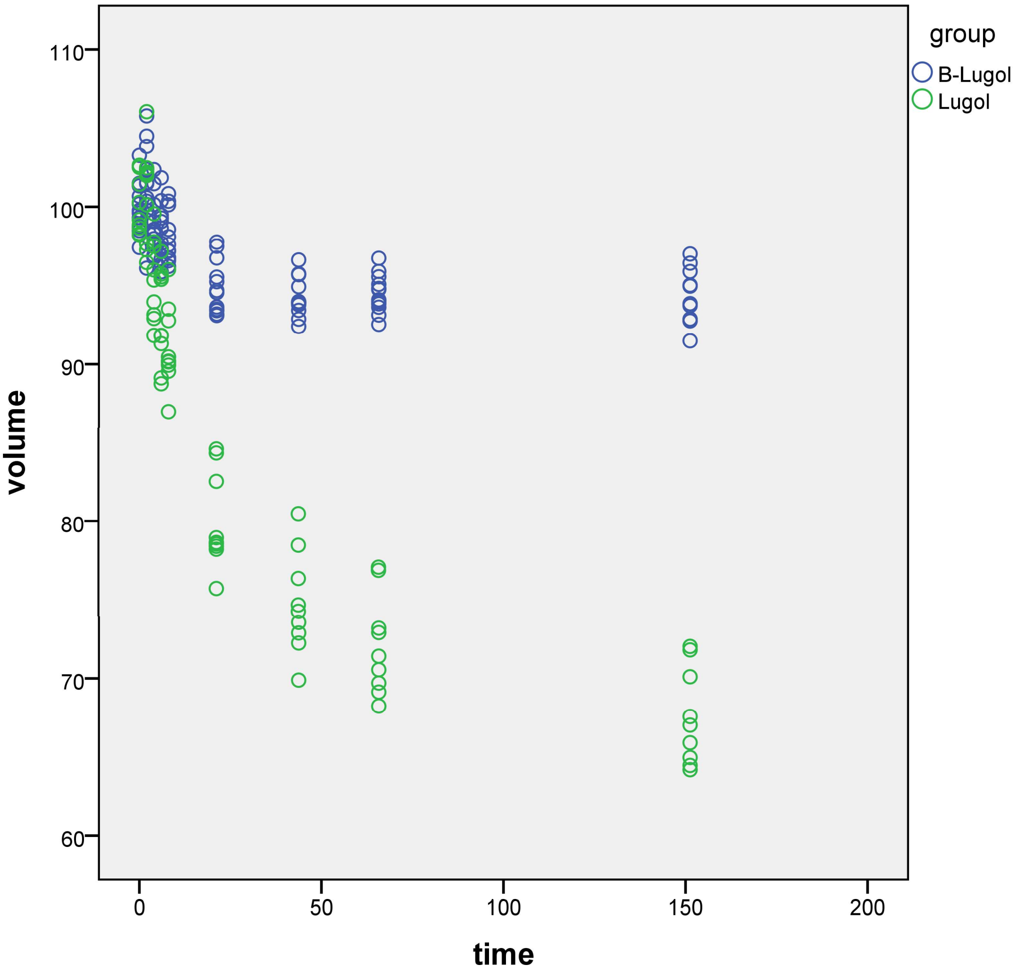

To determine the relation of pH and tissue volume in each group a linear regression analysis o f volume on pH was performed.

Regression

Model Summary[b]

group	Model	R	R Square	Adjusted R Square	Std. Error of the Estimate
B-Lugol	1	.758[a]	.574	.570	2.018435098
Lugol	1	.949[a]	.901	.899	3.885971269

a. Predictors: (Constant), pH

b. Dependent Variable: volume

ANOVA[a]

group	Model		Sum of Squares	df	Mean Square	F	Sig.
B-Lugol	1	Regression	576.874	1	576.874	141.596	.000[b]
		Residual	427.778	105	4.074		
		Total	1004.653	106			
Lugol	1	Regression	10823.196	1	10823.196	716.731	.000[b]
		Residual	1192.961	79	15.101		
		Total	12016.157	80			

a. Dependent Variable: volume

b. Predictors: (Constant), pH

Coefficients[a]

			Unstandardized Coefficients		Standardized Coefficients			95.0% Confidence ...
group	Model		B	Std. Error	Beta	t	Sig.	Lower Bound
B-Lugol	1	(Constant)	24.327	6.123		3.973	.000	12.187
		pH	10.610	.892	.758	11.899	.000	8.842
Lugol	1	(Constant)	44.556	1.609		27.690	.000	41.353
		pH	8.485	.317	.949	26.772	.000	7.854

Coefficients[a]

			95.0% Confidence ...
group	Model		Upper Bound
B-Lugol	1	(Constant)	36.467
		pH	12.378
Lugol	1	(Constant)	47.759
		pH	9.115

a. Dependent Variable: volume

This regression analysis shows that the 95% confidence interval of the slope of the relation between pH and tissue volume is overlapping between Lugol and B-Lugol indicating that the effect of pH on tissue volume is similar. And similar to the relation found in Experiment 1. The difference in intercept reflects the strong difference in shrinkage occurring during staining in the B-Lugol solution.

To complete the analysis as done in Experiment 1 a linear regression analysis of residual tissue volume on osmolarity was performed.

Regression

Variables Entered/Removed[a]

group	Model	Variables Entered	Variables Removed	Method
B-Lugol	1	Osmol[b]	.	Enter
Lugol	1	Osmol[b]	.	Enter

a. Dependent Variable: Unstandardized Residual

b. All requested variables entered.

Model Summary[b]

group	Model	R	R Square	Adjusted R Square	Std. Error of the Estimate
B-Lugol	1	.355[a]	.126	.110	1.68523709
Lugol	1	.099[a]	.010	-.013	2.76928541

a. Predictors: (Constant), Osmol

b. Dependent Variable: Unstandardized Residual

ANOVA[a]

group	Model		Sum of Squares	df	Mean Square	F	Sig.
B-Lugol	1	Regression	23.289	1	23.289	8.200	.006[b]
		Residual	161.881	57	2.840		
		Total	185.170	58			
Lugol	1	Regression	3.297	1	3.297	.430	.516[b]
		Residual	329.764	43	7.669		
		Total	333.062	44			

a. Dependent Variable: Unstandardized Residual

b. Predictors: (Constant), Osmol

Coefficients[a]

group	Model		Unstandardized Coefficients		Standardized Coefficients	t	Sig.	95.0% Confidence ...
			B	Std. Error	Beta			Lower Bound
B-Lugol	1	(Constant)	-15.866	5.380		-2.949	.005	-26.639
		Osmol	.027	.009	.355	2.864	.006	.008
Lugol	1	(Constant)	3.550	7.293		.487	.629	-11.159
		Osmol	-.016	.024	-.099	-.656	.516	-.064

Coefficients[a]

group	Model		95.0% Confidence ... Upper Bound
B-Lugol	1	(Constant)	-5.093
		Osmol	.046
Lugol	1	(Constant)	18.258
		Osmol	.033

a. Dependent Variable: Unstandardized Residual

Residuals Statistics[a]

group		Minimum	Maximum	Mean	Std. Deviation	N
B-Lugol	Predicted Value	-1.3562479	1.1883650	-.4734792	.63366109	59
	Residual	-3.14393616	3.90013909	.00000000	1.67064602	59
	Std. Predicted Value	-1.393	2.623	.000	1.000	59
	Std. Residual	-1.866	2.314	.000	.991	59
Lugol	Predicted Value	-2.1209779	-.8364543	-1.2249444	.27375139	45
	Residual	-6.12549114	4.02165127	.00000000	2.73763539	45
	Std. Predicted Value	-3.273	1.419	.000	1.000	45
	Std. Residual	-2.212	1.452	.000	.989	45

a. Dependent Variable: Unstandardized Residual

The linear regression analysis of residual tissue volume on osmolarity showed a small, but significant, positive effect of osmolarity on tissue volume. The slightly increasing osmolarity, despite the already very hypertonic nature of B-Lugol seems to counteract some of the shrinkage caused be the small decrease in pH.
